# Recurrent aphthous stomatitis information on TikTok: satisfactory or substandard?

**DOI:** 10.1007/s11845-025-04040-0

**Published:** 2025-09-02

**Authors:** Barry Patton, Anish Madan Lal, Richeal Ni Riordain

**Affiliations:** Department of Oral Medicine, Cork University Dental School & Hospital, University College Cork, Wilton, T12 E8YV Cork Ireland

**Keywords:** Aphthous, Medicine, Oral, RAS, Recurrent, Stomatitis, Ulcers

## Abstract

**Background:**

Social media is increasingly utilised as a means of disseminating oral health information. Recurrent aphthous stomatitis (RAS) is the most common ulcerating condition of the oral mucosa.

**Aim:**

The aim of this study was to evaluate the quality of TikTok videos concerning RAS.

**Material and methods:**

TikTok searches were performed using the terms “canker sores/#cankersores” and “aphthous ulcers/#aphthousulcers” and arranged by popularity. Videos were analysed and categorised into healthcare professionals (HCPs), non-healthcare professionals (non-HCPs) and other. Video quality was assessed using the Global Quality Score (GQS), modifed DISCERN (mDISCERN) and Patient Education Materials Assessment Tool (PEMAT).

**Results:**

Eighty-three videos were analysed. Overall, the videos were of poor quality with mean GQS and mDISCERN scores of 2.21 and 1.74 respectively. PEMAT understandability and actionability scores averaged 76.43% and 68.47% respectively. Videos uploaded by HCPs were generally of higher quality than others; this difference was statistically significant (*p* < 0.001). Weak positive correlation was noted between video engagement and quality (*p* < 0.05).

**Conclusion:**

The quality of videos examined was generally poor. It is important that social media users exert caution when utilising TikTok as a means of educating themselves about oral health. Additionally, healthcare professionals must be aware of health misinformation on social media, as it may negatively influence patient outcomes.

## Introduction

The advent of social media has ricocheted throughout all aspects of society and has become an increasingly visible platform for the delivery and consumption of healthcare-related information [1]. It is a common application through which younger adults receive active and passive education regarding their health, and for many it is now the primary method with which they research potential health issues [2]. This has progressed to the extent that many healthcare agencies and hospitals now actively seek to engage and inform populations en masse through social media-driven healthcare information campaigns [3].

Recurrent aphthous stomatitis (RAS) is a chronic oral condition characterised by repeated episodes of aphthous ulceration affecting the oral mucosa, primarily the non-keratinising mucosa of the buccal and labial tissues and tongue [4]. The ulcers are frequently referred to colloquially as “canker sores.” It is common, affecting between 20 and 25% of the world’s population, with a slightly higher prevalence in developed countries. The onset of RAS is typically between 10 and 19 years of age with a slight female predilection and becomes less common with advancing age [5]. While its aetiology is largely idiopathic, the putative factors posited are multifactorial, with psychosocial stress frequently identified as a trigger in developed countries, for example students around exam time [6].

The social media platform TikTok is currently the world’s second most popular social media application, behind Instagram. It is considered to be well suited to the dissemination of healthcare information due to its audio-visual format [7]. TikTok gained increased popularity and visibility in a healthcare context during the Covid-19 pandemic due to its widespread dissemination of both factual and erroneous context [8]. This resulted in many public health agencies indicating the potential importance of TikTok as a means of delivery of health-related information in the future [9].

Owing to the typical demographic affected by RAS, this study aimed to examine the quality of content currently present on TikTok given that the majority of users of this platform are in either the 18–24 or 25–34 age range (36.2% and 33.9% respectively) [10]. It aimed to examine the quality of videos delivered by both healthcare and non-healthcare professionals using validated quality assessments.

## Materials and methods

### Ethical considerations

All information was obtained from publicly released videos on TikTok, and non of the data involved personal privacy concerns. Therefore, no ethics review was needed.

#### Video collection

Two searches were performed on TikTok with the keywords “canker sores/#cankersores” and “aphthous ulcers/#aphthous ulcers” on the 1 st of August 2024. To avoid bias from personalised recommendations, personalised feeds and searches were turned off. Videos were ranked according to popularity on TikTok, with the number of likes being a surrogate for popularity. Eighty-three videos were selected based upon the inclusion criteria: videos in the English language pertaining to recurrent aphthous stomatitis that dealt with one or more of the following: epidemiology, aetiology, management, clinical presentation or course of the condition. Exclusion criteria comprised videos that did not meet the aforementioned criteria regarding aphthous ulceration, videos unrelated to aphthous ulceration and duplicate videos from the same content creators. The number of topics each video covered was documented on Microsoft Excel, with many videos covering multiple topics.

#### Video characteristics

Video attributes such as duration, views, likes, shares and comment count were systematically documented. A study specific data extraction document was generated using Microsoft Excel—video URL and attributes were recorded.

#### Video review and categorization

Two authors (BP and AML) independently reviewed the videos, and the final scores were averaged. The videos sourced were categorised by the uploader type: “healthcare professionals (HCPs)”, “non-healthcare professionals (non-HCP) sharing their personal experience” and “other”, which primarily comprised organisations and companies promoting products through RAS education. Distinction between HCPs and non-HCPs was made by evaluation of the TikTok channel disseminating the information and whether the channel and/or the content creator was purporting or verified to be a qualified healthcare professional (e.g. dentist) or representing a healthcare organisation.

#### Quality assessment

Again, two authors independently assessed the quality of the 83 videos between 2nd and 7th August 2024.

Global Quality Score (GQS), modified DISCERN (mDISCERN) tool and Patient Education Materials Assessment Tool (PEMAT) were utilized to evaluate the video quality [11–13].

The GQS is a subjective 5-point scale described in Table 1, it helped assess overall video quality, ranging from poor (1) to excellent (5). This scale was introduced in 2007 by a group of colleagues who aimed to review patient inflammatory bowel disease information resources on the world wide web [11]. Apart from the quality of the information, the GQS also considers the flow of the information. Although originally designed to evaluate the quality of written content, the flexibility of the scale makes it possible for it to be used to analyse video quality.

**Table 1:** Global quality score (GQS)

Score	Global Score Description
1	Poor quality, poor flow of the site, most information missing, not at all useful for patients
2	Generally poor quality and poor flow, some information listed but many important topics missing, of very limited use to patients
3	Moderate quality, suboptimal flow, some important information is adequately discussed but others poorly discussed, somewhat useful for patients
4	Good quality and generally good flow, most of the relevant information is listed, but some topics not covered, useful for patients
5	Excellent quality and excellent flow, very useful for patients

The mDISCERN was derived from the DISCERN tool and it helps measure the reliability of the videos. The DISCERN tool was developed in 1999; it consists of 15 questions that focus on various aspects of health information such as clarity and accuracy [12]. Each question is scored on a scale of 1 to 5 and the scores from the 15 questions are used generate an overall score that helps assess information quality and reliability. The higher the score, the higher the reliability and quality of the content. The mDISCERN is an abridged version of the DISCERN tool; it consists of 5 questions, with each question scored either 1 for “yes” or 0 for “no”. The scores are added, with higher scores corresponding to higher reliability. Details of the mDISCERN are provided in Table 2.

**Table 2:** Modified DISCERN (mDISCERN) tool

mDISCERN	
1. Is the aim clear, concise and understandable?	
2. Are valid sources cited? (from valid studies, dentists, or oral medicine specialists)?	
3. Is the information provided balanced and unbiased?	
4. Are additional sources of information listed for patient reference?	
5. Does the video address areas of controversy/uncertainty?	

The PEMAT was designed with the aim of assessing the quality of patient education materials and ensuring they are both understandable and actionable [13]. There are 2 versions of the PEMAT; PEMAT-P for printable materials and PEMAT-A/V for audio-visual materials. PEMAT-A/V was used in this study, it consists of 17 questions, with 13 representing the understandability (PEMAT-U) of health information and 4 evaluating the actionability (PEMAT-A) of the recommendations given by the videos. The questions are scored as “agree = 1, disagree = 0 and N/A”. A score of N/A is given if a question does not hold relevance in the context of the video. For example, one of the questions asks: whether the material uses illustrations and photographs that are clear and uncluttered? However, if no photographs or illustrations are used in the video, a score of “N/A” can be given. The total scores for the understandability and actionability sections are calculated as “Total points/Total possible points X 100”. Higher scores indicated better performance in each of the respective sections.

The final GQS, mDISCERN and PEMAT scores were an average of the score between the 2 authors.

The above tools have been validated by previous studies, particularly in the context of social media platforms [14–17].

#### Statistical analysis

IBM SPSS version 29.0.2.0 was used to analyse the data. Quantitative data (video duration, total views, likes, shares, comments, GQS, mDISCERN scores, PEMAT scores) was tested for normality using the Shapiro–Wilk test, and it was shown that the parameters did not represent a normal distribution (*p* < 0.001). Weighted Cohen’s kappa (κ) was used to measure agreement between the 2 raters for ordinal scores like the GQS [18]. For continuous scoring systems such as the mDISCERN and PEMAT the intraclass correlation coefficient (ICC) was used to measure inter-rater reliability. The κ and ICC values were interpreted as follows: κ/ICC > 0.8 indicated excellent agreeability; 0.6 < κ/ICC ≤ 0.8 suggested substantial agreement; 0.4 < κ/ICC ≤ 0.6 signified moderate agreement; and κ/ICC ≤ 0.4 was indicative of poor agreement [19]. A value of *p* < 0.05 was considered statistically significant.

The comparison among the three different uploader groups (HCP, Non-HCP personal experience and other) for variables with nonnormal distribution was analysed using the Kruskal–Wallis test. If the test yielded a statistically significant result for a particular variable, a post hoc Dunn’s test was conducted so pairwise comparisons can be made. A value of *p* < 0.05 was considered statistically significant.

To assess the relationship between audience interaction (total views, likes, shares, comments) and video quality (GQS, mDISCERN, and PEMAT scores), Spearman’s rank correlation coefficient (ρ) was used, with ρ > 0 denoting a positive relationship and ρ < 0 indicating a negative relationship. The strength of the correlation (ρ) was classified as follows: |ρ|≤ 0.2 represented no relationship; 0.2 <|ρ|≤ 0.4 implied a weak relationship; 0.4 <|ρ|≤ 0.6 indicated a moderate relationship; 0.6 <|ρ|≤ 0.8 suggested a strong relationship; and |ρ|> 0.8 denoted a very strong relationship. A value of *p* < 0.05 was considered statistically significant. Additionally, to test for consistency in the different methods used to assess video quality (GQS, mDISCERN, PEMAT) a Spearman’s rank correlation test was performed among them.

## Results

### Video characteristics

**Fig. 1:**
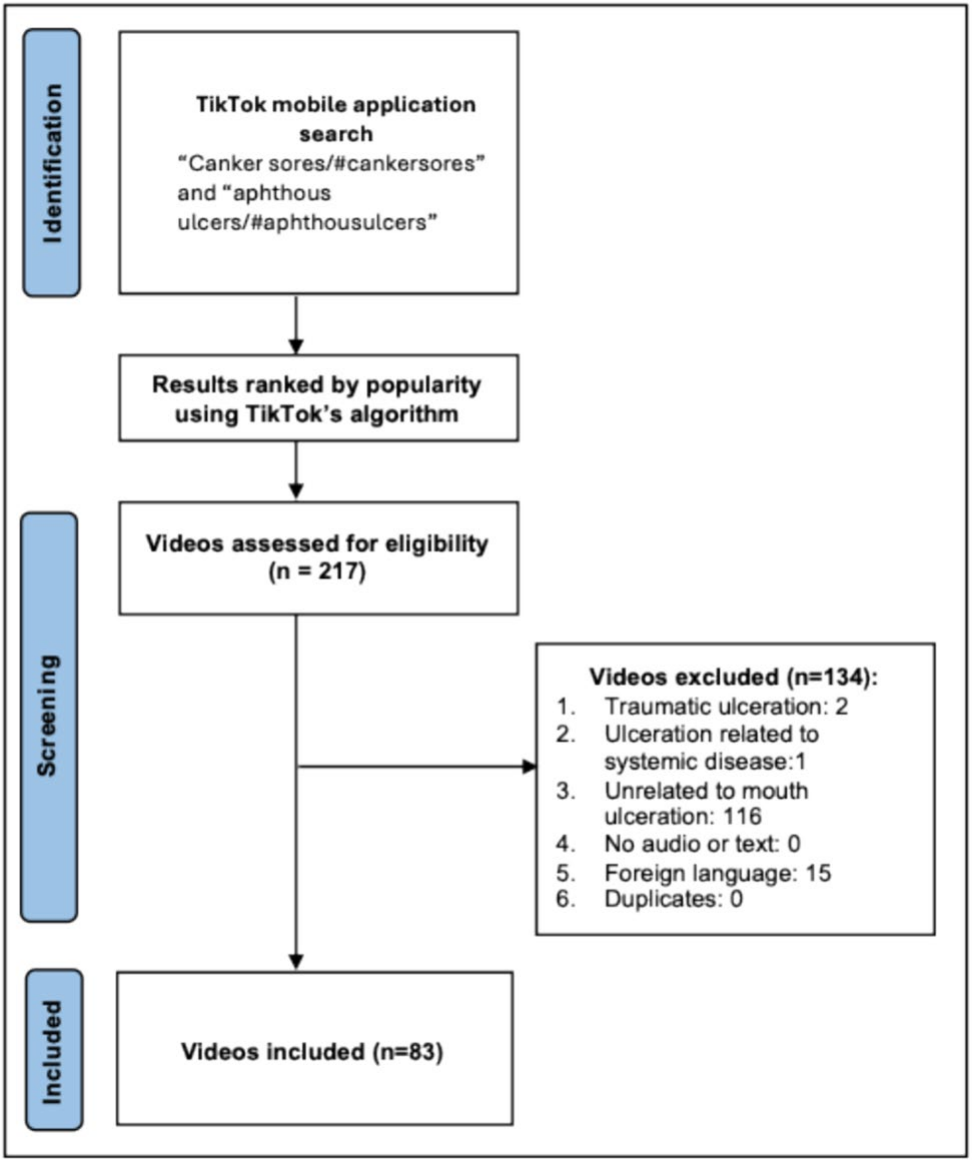
Search strategy for videos on RAS

The study included 83 videos after excluding non-relevant content (Fig. 1). The videos had a total of 50,193,320 views. Forty-one videos (49%) were uploaded by “HCPs”, 30 videos (36%) were personal experience videos uploaded by “non-HCPs” and 12 videos (14%) came under the category of “other”. An overwhelming majority of the videos discussed about management of the condition (75 videos), followed by aetiology (42 videos), clinical course/presentation (14 videos) and epidemiology (2 videos). Among the 83 videos, there was an average of 604,739 views (range, 522–8,600,000), 16,783 likes (range, 6–241,900), 1396 shares (range, 0–35,500) and 212 comments (range, 0–2390) per video. The average video length was 38.51 s (range, 6.0–160). Across all videos, the average GQS score was 2.21 (range, 1–5), average mDISCERN score was 1.74 (range, 0–3), average PEMAT-A/V understandability was 76.83% (range, 33.33–100%), average PEMAT-A/V actionability was 68.47% (range, 0–100%). These results demonstrate that the videos uploaded were generally of poor quality. The video characteristics in further detail are shown in Table 3.

**Table 3:** All video characteristics

Video Characteristics (*n*_t_=83)	
Mean Views *(SD, range)*	604,739 (1,389,915, 522–8,600,000)
Mean Likes *(SD, range)*	16,783 (39,642, 6–241,900)
Mean Shares *(SD, range)*	1,396 (4,937, 0–35,500)
Mean Comments *(SD, range)*	212 (442, 0–2,390)
Duration *(SD, range)*	38.51 (30.05, 6–160)
GQS *(SD, range)*	2.21 (0.91, 1–5)
mDISCERN *(SD, range)*	1.74 (0.81, 0–3)
PEMAT-A/V Understandability *(SD, range)*	76.83 (14.4, 33.33–100)
PEMAT-A/V Actionability *(SD, range)*	68.47 (27.42, 0–100)

### Inter-rater reliability

The reliability assessment of the video quality measured between the 2 authors demonstrated a strong level of agreement for GQS (κ 0.807, 95% CI: 0.714–0.9, *p* < 0.001), substantial agreement for mDISCERN (ICC 0.697, 95% CI: 0.568–0.793, *p* < 0.001), moderate agreement for PEMAT-U (ICC 0.583, 95% CI 0.421–0.709, *p* < 0.001) and moderate agreement for PEMAT-A (ICC 0.551, 95% CI 0.381–0.685, *p* < 0.001). Overall, the raters had a good level of agreement between them.

### Video quality

No statistically significant differences (*p* > 0.05) in engagement metrics (views, likes, shares and comments) were seen among the different uploader groups (HCP, non-HCP and other). However, notable differences in video quality were found, with GQS, mDISCERN and PEMAT understandability scores all showing significant differences among the groups (*p* < 0.05). In contrast, PEMAT actionability scores had no significant differences. Pairwise comparisons among the GQS, mDISCERN and PEMAT-U scores revealed that videos uploaded by health care professionals (HCPs) and organisations (other) had significantly higher scores compared to videos uploaded by non-healthcare professionals sharing their personal experience. Videos uploaded by HCPs had a higher understandability (PEMAT-U scores) compared to the rest of the groups. Video quality and characteristics among different groups of uploaders is shown in Table 4.

**Table 4:** Video characteristics by uploader type

Video Characteristics	HCP (*n*_1_=41)	Non-HCP personal experience (*n*_2_=30)	Other (*n*_3_=12)	P	P_H-N_	P_N-O_	P_H-O_
Mean Views *(SD, range)*	547,928 *(993,250, 593–4,000,000)*	558,994* (1,633,187, 645–8,600,000)*	913,207 *(1,913,169, 522–5,800,000)*	0.213^a^			
Mean Likes *(SD, range)*	14,309 *(30,221, 8–136,300)*	16,854 *(36,379, 12–128,700)*	25,060 *(69,334, 6–241,900)*	0.193^a^			
Mean Shares *(SD, range)*	372 *(846, 0–5,145)*	2,108 *(7,015, 0–35,500)*	3,117 *(6,389, 0–20,400)*	0.447^a^			
Mean Comments *(SD, range)*	218* (426, 0–1,929)*	167 *(347, 2–1,582)*	304 *(681, 0–2,390)*	0.517^a^			
Duration *(SD, range)*	33.8 *(27.61, 10–153)*	45.70 *(37.11, 6–160)*	36.58 *(10.07, 20–52)*	0.152^a^			
GQS *(SD, range)*	2.73 *(0.84, 1–5)*	1.5 *(0.51, 1–2.5) *	2.21 *(0.69, 1–3)*	**<0.001**^a^	**<0.001**^b^	**0.009**^b^	0.106^b^
mDISCERN *(SD, range)*	2.22 *(0.67, 1–3)*	1.1 *(0.5, 0–2)*	1.71 *(0.78, 1–3)*	**<0.001**^a^	**<0.001**^b^	**0.029**^b^	**0.047**^b^
PEMAT-A/V Understandability *(SD, range)*	82.37 *(11.19, 50–100)*	67.34 *(14.69, 33.33–92.86)*	81.60 *(11.71, 53.57–92.86)*	**<0.001**^a^	**<0.001**^b^	**0.005**^b^	0.291^b^
PEMAT-A/V Actionability *(SD, range)*	73.98 *(28.88, 0–100)*	61.67 *(25.58, 0–100)*	66.67 *(24.62, 16.67–100)*	0.078^a^			

*P*_H-N_ HCP versus Non-HCP, *P*_N-O_ Non-HCP versus Other, *P*_H-O_ HCP versus Other. Bold text means the *P-*value<0.05

^a^ Kruskal–Wallis test

^b^ Dunn’s test

### Correlation analysis

Correlation analysis is shown in Table 5. No strong relationships were found between the video quality and audience engagement. GQS and PEMAT-U scores had weak to moderate positive relationships with the audience interaction. A strong positive relationship was found among the GQS, mDISCERN and PEMAT-U scores indicating consistency in how these tools evaluate content. This also suggests that higher quality videos are more understandable. A weak positive relationship was observed among GQS, PEMAT-U and PEMAT-A, suggesting a modest connection between understandability and actionability.

**Table 5:** Spearman’s rank correlation

		Views	Likes	Shares	Comments	Duration	mDISCERN	PEMAT-A/V Understandability	PEMAT-A/V Actionability
GQS	***ρ***	0.288**	0.218*	0.197	0.261*	−0.057	0.842**	0.624**	0.248*
	***P*** value	**0.008**	**0.048**	0.074	**0.017**	0.607	**<0.001**	**<0.001**	**0.024**
mDISCERN	***ρ***	0.161	0.092	0.093	0.111	−0.122		0.564**	0.191
	***P*** value	0.146	0.406	0.401	0.317	0.273		**<0.001**	0.083
PEMAT-A/V Understandability	***ρ***	0.250*	0.181	0.223*	0.202	0.008			0.396**
	***P*** value	**0.023**	0.101	**0.043**	0.068	0.946		.	**<0.001**
PEMAT-A/V Actionability	***ρ***	0.103	0.110	0.182	0.062	0.095			
	***P*** value	0.356	0.323	0.099	0.575	0.393			

Bold text means the *P-*value<0.05

** Correlation is significant at the 0.01 level (2-tailed)

* Correlation is significant at the 0.05 level (2-tailed)

## Discussion

The results indicate that a generalised dearth of quality, evidence-based information is available on TikTok regarding recurrent aphthous stomatitis (RAS). Firstly, the majority of the videos (75 out of 83) that met inclusion criteria addressed the management of RAS. In other TikTok content analysis studies, videos uploaded by HCPs did have a higher quality compared to other uploaders; however, videos discussing management did not always form the bulk of the data [14, 17]. The reason for the focus on management may be attributed to the fact that RAS presents acutely and a prompt solution for symptom management may be the primary focus of searches on TikTok and other social media platforms, as opposed to an explanation of the underlying pathophysiology. Therefore, creators may prioritise content that deals with treatment in order to address the viewers’ concern over their active symptoms. Currently, topical therapies are the mainstay of treatment, including the use of corticosteroid mouth rinses, gels and sprays containing local anaesthetic compounds and covering medicaments, for example the emollient Orabase [20]. In addition, mouthwashes containing chlorhexidine gluconate, a biguanide antiseptic available over-the-counter, have also demonstrated efficacy in the reduction of symptoms duration and severity [21].

Owing to its proposed irritative effect on the oral mucosa, avoiding toothpastes containing sodium lauryl sulphate (SLS) is also a recommended approach in the management of RAS [22]. SLS in an anionic surfactant frequently present in dentifrices which produces a foaming effect in toothpaste through decreasing water surface tension, as well as having an antimicrobial action via its wetting ability allowing cell wall penetration and interaction with cell membrane constituents and lipids [23]. However, these actions can promote shedding of the squamous epithelium and may lead to dryness of the protective layer of the oral mucosa, thereby facilitating and/or perpetuating ulcer development [24]. A substantial proportion of the videos examined postulated SLS as a defining cause of RAS and switching to an SLS-free toothpaste would promote RAS resolution. The literature suggests this is inaccurate; a 2012 randomised control trial found that, while SLS-free compounds helped accelerate the reparative healing process and reduced pain, they did not have any significant on the initial development or number of ulcers [25].

Limited evidence is available as to the benefit of systemic agents in the management of RAS. Generally, topical and preventative strategies are recommended in the first instance before systemic options are considered, and a 2012 Cochrane review found that no single therapy was effective as a treatment modality and, moreover, that any benefits of a single drug was likely a reflection of its success on an individual basis and not generalisable to wider populations [26]. Commonly utilised medications in the management of RAS include colchicine, short courses of systemic corticosteroids and non-steroidal anti-inflammatory drugs (NSAIDs). Colchicine is a potent anti-inflammatory agent which acts via cytoskeletal disruption through inhibition of beta-tubulin polymerisation [27]. It is frequently used in the treatment and prophylaxis of gout. An off-label trial can be recommended in patients with persistent and active disease and has been demonstrated to produce a clear improvement in symptoms in those who tolerate the drug [28]. It is associated with numerous adverse effects which often preclude its administration, namely nausea and diarrhoea, which affect up to 45% of patients [29, 30].

Furthermore, systemic corticosteroids may be indicated in treatment-resistant disease and/or if other systemic agents do not produce an improvement. Typically, doses of 10–30 mg of prednisolone for a duration of up to 1 month has been shown to reduce pain and duration of ulceration; however, longer-time use of 5 mg prednisolone for up to three months has shown comparable efficacy to that of colchicine [31]. A host of other systemic agents are possible in RAS management, including pentoxifylline, dapsone, sucralfate and cyclosporine, none of which were discussed as options in the TikTok videos examined for this study [30].

Numerous alternative proposed treatment approaches recurred throughout the videos, for example the use of alum powder featured prominently. It is a colourless, odourless crystal known as aluminium sulphate. It has traditionally been used in the application of herbal medicine techniques, and it is posited to contain anti-inflammatory and astringent properties which aid the resolution of aphthous ulceration. Few studies exist in the literature examining its therapeutic benefits in the context of RAS, but those that do suggest some improvement in both symptom duration and severity upon application of either its salt or solution form directly onto affected sites [32, 33]. Several novel therapies have been proposed in the treatment of RAS, however. For example, the implementation of a single application of silver nitrate as a form of chemical cauterisation has demonstrated significant reduction in acute pain associated with aphthous ulceration [34]. In addition, the use of a carbon dioxide laser directed at affected areas has demonstrated some effectiveness at sustained analgesia following administration [35].

Despite being a crucial aspect to the understanding of the condition, just over half of videos viewed (42 out of 83) addressed the aetiopathogenesis of RAS. In relation to videos which discussed aetiology, sodium lauryl sulphate was the most frequently cited single aetiological factor. However, the literature has demonstrated multiple other risk factors underlying RAS development. Significantly, haematinic deficiencies—namely iron, vitamin B12 and folic acid—have often been cited as possible underlying causes of RAS [36]. A 2015 meta-analysis found deficiencies of these substrates were significantly higher among patients with RAS when compared to control populations [37]. Other recognised risk factors, such as psychosocial stress, genetic predisposition and recent smoking cessation, were only occasionally mentioned, or in some cases omitted, from the videos examined, highlighting potential deficits in the communication of the aetiopathogenesis of RAS, in particular given that in most instances the aetiology is idiopathic [38].

The information needs of patients has assumed heightened importance in recent decades with the increasingly shared responsibility for healthcare management, with studies historically and consistently demonstrating that patients feel they often seek more information than is given in a typical consultation, and that they generally formulate most questions to pose to the clinician after the appointment is over [39]. Measures on information needs are often absent in the case of widespread common chronic conditions with the need for more psychometric studies to evaluate existing needs [40]. There is also evidence to suggest the implementation of knowledge tests, for example true-or-false questions, may be beneficial both in terms of guiding delivery of education content and as a means of surveillance of the overall patient education process [41].

This study demonstrated a statistically significant difference in the quality of information contained in videos crafted by healthcare professionals when compared to those by non-healthcare professionals conveying personal experiences. Misinformation in health-related content has been a persistent issue following the advent of social media and has increased over time [42]. This problem has particularly accelerated from the onset of the Covid-19 pandemic, a time when the use of social media platforms to communicate vital and urgent public health information from official sources saw a concurrent rise in the spread of intentional misinformation from non-reputable sources [43]. Only 3.6% of videos provided suitable links to resources for further information on the topic or clearly cited the sources they used, also highlighting potential discrepancies in the quality of information communicated. Despite the disparate quality, no significant difference in audience engagement was noted between HCP and non-HCP videos. Previous studies examining similar topics have noted that video length (particularly under 60 s) and videos that reported on the domains of risk factors, management and outcomes were more likely to be shared generally and therefore boost audience engagement [44].

The results of the study are broadly in line with similar studies examining patient information in relation to other conditions. For example, a recent review on web-based material pertaining to osteosarcoma found that the educational resources scored poorly in terms of readability, understandability and actionability as per the PEMAT metrics [45]. Similarly, information for patients living with chronic diseases such as chronic obstructive pulmonary disease also displayed uncertainty regarding information quality, with considerable variability among patients themselves in terms of their ability to distinguish high quality from low quality source material [46]. Factors influencing patient ability to achieve this included disease severity, health-related quality of life and degree of background baseline knowledge regarding their condition.

A recurring theme regarding delivery of patient information in these contexts is that of desire on behalf of the patient to actually receive this information—this can be influenced by disparate factors such as timing, patient preparedness to learn, barriers to receiving information such as literacy and evolving priorities over time [47]. It would indicate that due consideration to these aspects would be prudent when endeavouring to communicate information regarding RAS.

## Conclusion

In the steadily expanding and diversifying landscape of social media, the results of this study suggest a heightened awareness is necessary on the part of healthcare professionals active on social media platforms to ensure comprehensive and accurate information on the aetiology and management of RAS.

## Data Availability

The data that supports the findings of this study are available from the corresponding author, Barry Patton, upon reasonable request.
